# Adsorption of Sr^2+^ from Synthetic Waste Effluents Using Taiwan Zhi-Shin Bentonite

**DOI:** 10.3390/ijms26115298

**Published:** 2025-05-30

**Authors:** Yihui Lin, Yuhan Li, Yating Yang, Po-Hsiang Chang

**Affiliations:** 1 Department of Geography, Hanshan Normal University, Chaozhou 521041, China; 20190031@hstc.edu.cn; 2 College of Resources and Environment, Fujian Agriculture and Forestry University, Fuzhou 350002, China; yuhan200203@163.com

**Keywords:** strontium, Taiwan Zhi-Shin bentonite, adsorption mechanism, cation exchange, waste liquid treatment

## Abstract

This study investigated strontium (Sr^2+^) adsorption by Taiwan Zhi-Shin bentonite (cation exchange capacity (CEC): 80–86 meq 100 g^−1^) using Sr(NO_3_)_2_-simulated nuclear waste. Kinetic analysis revealed pseudo-second-order adsorption kinetics, achieving 95% Sr^2+^ removal within 5 min at pH 9. Isothermal studies showed a maximum capacity of 0.28 mmol g^−1^ (56 meq 100 g^−1^) at 15 mmol L^−1^ Sr^2+^, accounting for 65–70% CEC and fitting the Freundlich model. Cation exchange was the dominant mechanism (84% contribution), driven by Sr^2+^ displacing interlayer Ca^2+^. Alkaline conditions (pH > 9) enhanced adsorption through improved surface charge and electrostatic attraction. Thermodynamic studies demonstrated temperature-dependent behavior: increasing temperature reduced adsorption at 0.01 mM Sr^2+^ but increased efficiency at 10 mM. Na^+^ addition suppressed adsorption, aligning with cation exchange mechanisms. Molecular dynamics simulations identified hydrated Ca^2+^-Sr^2+^ water bridges interacting with bentonite via hydrogen-bonding networks. The material exhibits rapid kinetics (5 min equilibrium), alkaline pH optimization, and resistance to ion interference, making it suitable for emergency Sr^2+^ treatment. It shows promise as a cost-effective and good performing adsorbent for radioactive waste solutions.

## 1. Introduction

As a heavy metal element with high solubility and mobility, the radionuclide strontium (Sr^2+^) is prevalent in nuclear industry wastewater and contaminated aquatic environments. Due to chemical properties analogous to those of calcium and barium [1], Sr^2+^ readily infiltrates human bone tissue through ion exchange mechanisms and forms stable Sr_3_(PO_4_)_2_ compounds. These results showed prolonged bioaccumulation with limited metabolic elimination [2]. Functioning as a bone-seeking beta radiation emitter, Sr^2+^ induces cutaneous radiation injury [3] and has been shown to provoke severe pathologies including leukemia, osteosarcoma, and hematopoietic disorders (characterized by marked depletion of leukocytes, erythrocytes, and thrombocytes), constituting significant health hazards [4]. Notably, the radioisotope ^90^Sr^2+^ exhibits an extended half-life of 28.79 years [5] and demonstrates pronounced environmental persistence. The 2011 Fukushima Daiichi nuclear disaster in Japan, which released substantial quantities of radioactive Sr^2+^ into marine ecosystems, has heightened global concerns regarding Sr^2+^ contamination [6]. Presently, Sr^2+^ concentrations in water in Chinese cities average 360 ppb [7].

With the rapid development of the nuclear industry, radioactive wastewater discharge has emerged as a primary pathway for radionuclides to enter aquatic environments. The development of efficient Sr^2+^ removal technologies from water systems constitutes a critical research frontier in environmental science and engineering. Current mainstream techniques include ionic exchange [8], chemical precipitation [9], evaporation concentration [10], bioremediation approaches [11], photocatalysis [12], and adsorption [13] (Table S1). Nevertheless, these methodologies exhibit operational limitations under practical applications. For instance, the evaporation concentration demonstrates excessive energy demands and process-related risks including equipment corrosion and scaling [14]. Bioremediation approaches suffer from suboptimal efficiency and environmental dependency [15,16]. The regeneration effluents generated by ionic exchange systems pose secondary contamination risks [17,18], while membrane separation technologies are constrained by prohibitive operational costs and low resource recovery efficiency [19]. Consequently, the innovation of high-performance, cost-effective, and ecologically sustainable removal technologies has become a strategic priority in contemporary scientific investigations.

The adsorption method is recognized as one of the most promising technologies for radioactive Sr^2+^ removal, attributed to its operational simplicity, cost-effectiveness, and broad applicability. Recent research has explored diverse organic and inorganic adsorbents, including ion-exchange resins, lanthanide oxalate frameworks, natural minerals, graphene oxide composites, metal-organic frameworks (MOFs), polymeric hydrogels, and novel engineered sorbents, as documented in studies [20,21,22,23,24,25,26,27,28] (Table S2). Conventional adsorbents such as activated carbon were shown to exhibit constrained Sr^2+^ sorption capacities (e.g., 44.42 mg g^−1^), while existing studies frequently fail to specify critical operational parameters such as optimal adsorbent dosage, thereby impeding practical implementation [29]. Furthermore, the research and development cost of new adsorption materials is high, and the energy consumption is large, so it is difficult to popularize modified materials like Moloukhia et al. [30] on a large scale. Therefore, it is urgent to find a natural mineral material with low cost and excellent adsorption property.

Bentonite, as a layered silicate mineral consisting mainly of two layers of silico-oxygen tetrahedrons and one layer of alumino-oxygen octahedrons, has a high specific surface area and cation exchange capacity (CEC), capable of highly efficient adsorption of Sr^2+^ in water by cation exchange and electrostatic action [31]. As a bentonite resource with abundant reserves and low price [32], the unique layered structure and surface chemical properties of Taiwan Zhi-Shin bentonite show great potential in the field of radioactive Sr^2+^ adsorption [33,34]. Studies have shown that the adsorption performance of bentonite can be further improved after acidification or organic modification, which is significantly better than that shown by traditional adsorbents [35,36]. In addition, the metal sulfide of Na_2_Sn_2_S_7_ has been shown to be stable over a wide pH range and to have a strong resistance to ion interference, which is suitable for Sr^2+^ removal in complex water environments [37]. Taiwan is an island with three nuclear power plants, and its nuclear waste has been piling up on Lanyu Island (located in the southeast of Taiwan). The Zhi-Shin soil in this study is from Taitung, Taiwan, which is quite close to Lanyu Island, and this material has the characteristics of high efficiency and economical production. Studying its adsorption performance for Sr^2+^ is helpful for developing the buffering behavior of Taiwan Zhi-Shin bentonite against nuclear waste. It provides a potentially advantageous material for the disposal of nuclear waste in Taiwan and even around the world. Although bentonite has shown significant advantages in Sr^2+^ adsorption, it still faces some challenges in practical applications. For example, the presence of competing ions (e.g., Ca^2+^, Mg^2+^) may affect its selective adsorption on Sr^2+^ [38], and the adsorbed bentonite needs to be efficiently recycled or cured to avoid secondary pollution [39]. Therefore, future research should focus on modification optimization, dynamic adsorption properties, and actual wastewater treatment verification of bentonite in order to promote transformation from laboratory research to engineering applications.

In this study, Taiwan Zhi-Shin bentonite was used as the adsorbent, and the batch method was employed to study the adsorption properties of Taiwan Zhi-Shin bentonite on Sr^2+^ under different environmental conditions (time, concentration, temperature, pH, and ionic strength). Through characterization techniques such as X-ray diffraction (XRD), the impact of different pH levels and initial concentrations on adsorption performance was explored, and molecular simulation techniques were utilized to verify the respective adsorption mechanism. A systematic discussion on its adsorption performance and mechanism for Sr^2+^ in water was conducted. Based on the efficient adsorption technology of bentonite, solving the problem of Sr^2+^ pollution is of great significance. The aim is to provide an economical, efficient, and environmentally friendly solution for the treatment of radioactive wastewater.

## 2. Results

### 2.1. Adsorption Kinetics Experiment of Strontium (Sr^2+^)

**Figure 1:**
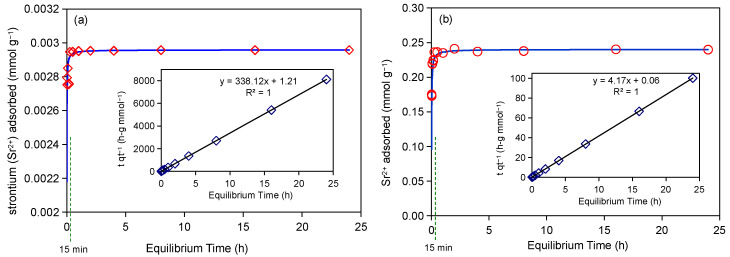
The adsorption kinetics of 0.1 mM-Sr (**a**) and 10 mM-Sr (**b**) on Taiwan Zhi-Shin bentonite. The solid line is pseudo-second-order fit to the observed data. The dashed green line represents the time when equilibrium was reached. Insert is the linear plot of the equation.

The adsorption kinetics data of Sr^2+^ using Taiwan Zhi-Shin bentonite, as illustrated in Figure 1, exhibited excellent agreement with a pseudo-second-order kinetic model (R^2^ = 1.000), a mechanism frequently observed in cation exchange-dominated adsorption systems [40]. It is worth noting that the system achieved adsorption equilibrium within 15 min under both 0.1 mmol L^−1^ (0.1 mM-Sr) and 10 mmol L^−1^ (10 mM-Sr) initial Sr^2+^ concentrations (Figure 1a,b), demonstrating that equilibrium time remained unaffected by increasing Sr^2+^ concentrations. This rapid adsorption behavior aligns with prior studies [41] and highlights distinctive advantages over other reported materials (Table S3). Specifically, Taiwan Zhi-Shin bentonite achieves equilibrium comparably fast to Na-bentonite (11–14 min) [13], implying that the interlayer structure of montmorillonite facilitates Sr^2+^ adsorption kinetics. However, unlike Na-bentonite, which exhibits reversible Sr^2+^ adsorption unsuitable for practical applications [42], Taiwan Zhi-Shin bentonite maintains irreversible binding. Its performance significantly surpasses slower adsorbents such as metal-organic framework/potassium nickel hexacyanoferra (MOF/KniFC) composites (45 min) [25], basic zeolites (6 h) [23], and sulfonylcalixarene-loaded XAD-7 resin (7 h) [20]. Furthermore, Taiwan Zhi-Shin bentonite operates effectively across a broad concentration range (0.1–10 mmol L^−1^), unlike basic zeolites, which excel only at low concentrations (50 mg g^−1^) [23]. These rapid adsorption properties, coupled with minimal pretreatment requirements (e.g., no acid activation or ion exchange) and lower cost compared to synthetic materials, position Taiwan Zhi-Shin bentonite as an ideal candidate for emergency remediation of sudden radionuclide contamination. The combined effects of its adsorption kinetics advantages and inherent interlayer structural features enabled efficient Sr^2+^ capture via cation exchange, thereby optimizing nuclear waste management strategies.

### 2.2. Adsorption Isotherm Experiment of Sr^2+^

**Figure 2:**
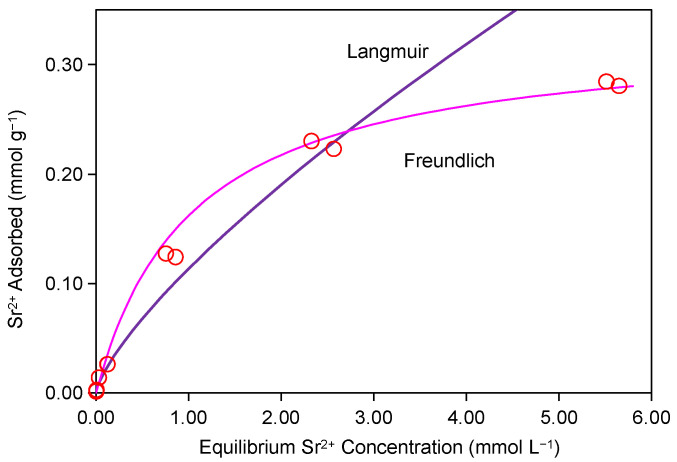
Adsorption of Sr^2+^ on Taiwan Zhi-Shin bentonite. The solid lines are the Langmuir and Freundlich fit to the observed data.

The adsorption isotherm data of Sr^2+^ on Taiwan Zhi-Shin bentonite were fitted using Freundlich and Langmuir models, with the Freundlich model (R^2^ = 0.96) displaying a significantly better agreement than the Langmuir model (Figure 2). This result revealed heterogeneous surface characteristics and a multilayer adsorption mechanism [43], further corroborated by the Freundlich parameter *n* = 1.196 (1 < *n* < 10) listed in Table 1. The value of *n* indicates a continuous increase in adsorption capacity with rising Sr^2^⁺ concentrations within the experimental range [44], achieving a maximum adsorption capacity of 0.28 mmol g^−1^ (24.53 mg g^−1^), which corresponds to 65–70% of its CEC. Although Na-bentonite [13], MOF/KNiFC composites [25], and basic zeolites [23] exhibited higher adsorption capacities (Table S2), their practical applicability is constrained by high costs and complex pretreatment requirements. In contrast, Taiwan Zhi-Shin bentonite demonstrates distinct advantages in engineering scenarios due to its natural abundance, absence of pretreatment needs, and operational simplicity. Previous mechanistic studies on Sr^2+^ adsorption by bentonite confirm cation exchange as the dominant adsorption mechanism [13,45]. It is worth noting that the adsorption capacity of Taiwan Zhi-Shin bentonite (24.53 mg g^−1^) was significantly lower than that of Na-bentonite (46 mg g^−1^) [13], a disparity attributed to differences in ion-exchange behavior between Ca-bentonite (Taiwan Zhi-Shin bentonite) and Na-bentonite. The smaller hydrated ionic radius of Na^+^ (1.16 Å) compared to Ca^2+^ (1.26 Å) and its lower ion-exchange energy barrier facilitate more efficient competitive substitution with Sr^2+^ [46], a phenomenon consistent with the established influence of exchangeable cation radii on ion-exchange efficiency in clay minerals [34]. Raw bentonite ore, predominantly composed of Ca-bentonite with impurities, requires Na₂CO₃ processing to produce pure Na-bentonite [47,48]. As a natural Ca-bentonite, Taiwan Zhi-Shin bentonite retains inherent ion-exchange limitations due to its unmodified state. Nevertheless, its direct utilization without chemical modification, coupled with abundant natural reserves and compatibility with industrial protocols, positions it as a preferred buffer material for nuclear waste disposal applications.

### 2.3. Experiments on Influencing Factors of Adsorption of Sr^2+^

Ion exchange constitutes the overarching framework governing Sr^2^⁺ adsorption, as evidenced by concomitant cation desorption patterns.

#### 2.3.1. Exchangeable Cation Desorption

**Figure 3:**
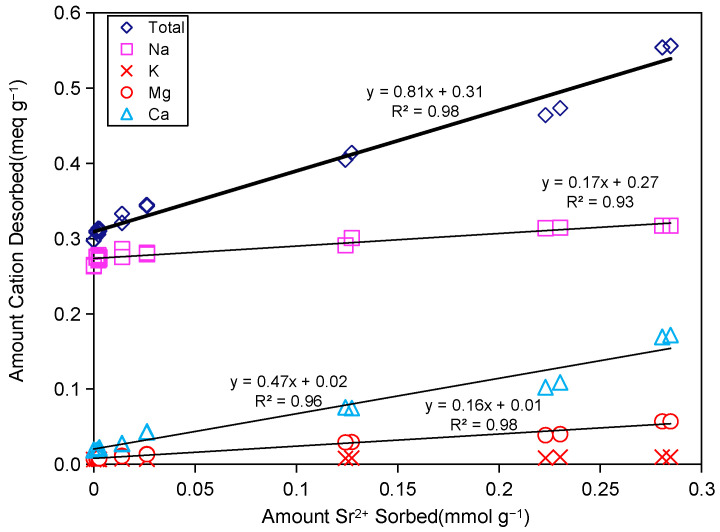
Desorption of metal cations from Taiwan Zhi-Shin bentonite as affected by the amount of Sr^2+^ adsorption.

Figure 3 illustrates the release of metal cations (Na^+^, K^+^, Ca^2+^, and Mg^2+^) accompanying the adsorption and interlayer incorporation of Sr^2+^ ions, with cation exchange identified as the primary mechanism governing Sr^2+^ adsorption in Taiwan Zhi-Shin bentonite. Within the entire application range of Sr^2+^ concentration, the molar ratio between the total amount of cation desorption and the adsorbed Sr^2+^ was close to 1:1 (with a slope of 0.81), proving that ion exchange plays an important role in the adsorption mechanism of Taiwan Zhi-Shin bentonite and Sr^2+^. This is consistent with the results of previous studies [13,34,38,45,49]. The interlayer cations of Taiwan Zhi-Shin bentonite were shown to be predominantly Ca^2+^ (dominant species), accompanied by minor Na^+^ and Mg^2+^ (see Table S4 for detailed composition). During Sr^2+^ adsorption, the concomitant cation release exhibited distinct patterns: K^+^ release remains negligible and largely independent of Sr^2+^ adsorption levels, whereas Na^+^ and Mg^2+^ release increased marginally. Notably, Ca^2+^ dominated the displacement process, accounting for 60.4% of the total released cations relative to the adsorbed Sr^2+^ concentration (Figure 3). This observation confirms cation exchange as the principal driver of Sr^2+^ uptake in Taiwan Zhi-Shin bentonite. A strong positive correlation exists between adsorbed Sr^2+^ quantities and released cation concentrations, demonstrating that Sr^2+^ adsorption directly triggers the displacement of native interlayer cations, particularly Ca^2+^. This preferential exchange likely stems from differences in charge density and hydration properties between Sr^2+^ and resident interlayer cations [50]. The observed behavior aligns with studies on silicoantimonate systems, where Ca^2+^ similarly mediated Sr^2+^ adsorption dynamics [28]. These findings not only elucidate the high Sr^2+^ adsorption capacity of bentonite but also advance the mechanistic understanding of clay minerals in regulating the immobilization and transport of heavy metal contaminants within engineered and natural environments.

#### 2.3.2. Influence of pH Value

**Figure 4:**
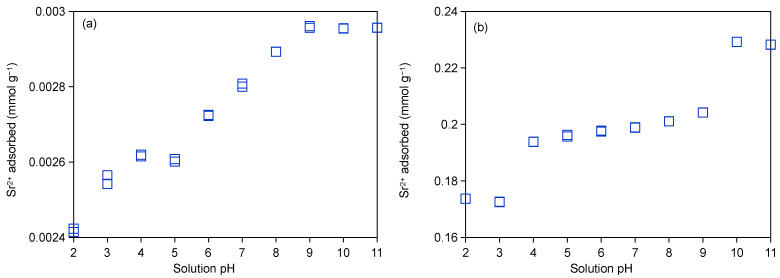
The 0.1 mM-Sr (**a**) and 10 mM-Sr (**b**) adsorption on Taiwan Zhi-Shin bentonite as affected by equilibrium solution pH.

**Figure 5:**
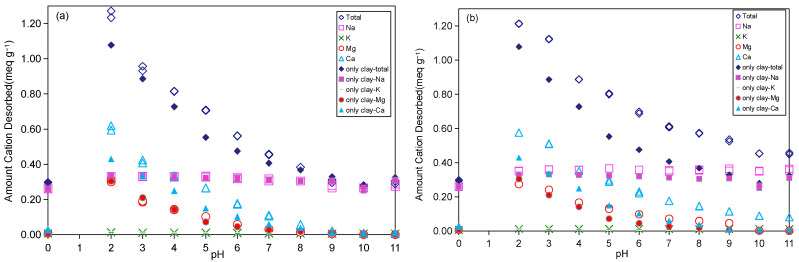
Desorption of metal cations from Taiwan Zhi-Shin bentonite as affected by the pH values under the amount of 0.1 mM-Sr adsorption (**a**) and 10 mM-Sr (**b**).

The pH-dependent adsorption characteristics of Sr^2+^ on Taiwan Zhi-Shin bentonite are presented in Figure 4a,b for 0.1 mM-Sr and 10 mM-Sr, respectively. Figure 5a,b further shows cation desorption patterns under corresponding conditions. A consistent upward trend in Sr^2+^ adsorption emerges with increasing pH across both concentrations, though mechanistic divergences arise depending on initial Sr^2+^ loading. At 0.1 mM-Sr, rapid adsorption saturation occurred due to minimal competitive interference, whereas the 10 mM-Sr system exhibited constrained adsorption enhancement attributable to Sr^2+^ hydrolysis effects and competitive adsorption with Na^+^, Mg^2+^, and Ca^2+^ (Figure 4b and Figure 5b). Speciation analysis confirmed Sr^2+^ as the dominant aqueous species between pH 1–11, with minor Sr(OH)^+^ formation, transitioning to Sr(OH)^+^ dominance at pH > 13 [51]. The point of zero charge (PZC = 9.2) critically modulated adsorption behavior: below pH 4, elevated H^+^ concentrations competed with Sr^2+^ for negatively charged surface sites, suppressing adsorption (Figure 4a,b). Progressive pH elevation (5–9) facilitated OH^-^-mediated outer-sphere complexation [52], enabling enhanced Sr^2+^ adsorption via electrostatic attraction in the electrical double layer (Figure 4a). For the 10 mM-Sr system, however, limited adsorption gains in this pH range reflect persistent cation competition (Figure 4b and Figure 5b). Above pH 9, Ca^2+^ precipitation [53] alleviated competitive effects, driving substantial Sr^2+^ uptake (Figure 4a). At pH 10, co-existing Sr^2+^ and Sr(OH)^+^ species synergistically enhanced adsorption through dual electrostatic interactions (Figure 4b) [54], though adsorption plateaus at pH 11 due to renewed competition from Na^+^ and residual Ca^2+^ occurred (Figure 5b). The pH-modulated adsorption trends ultimately reflect perturbations in the ion exchange equilibrium established in Section 2.3.1. The adsorption behavior was not only affected by the pH and initial concentration of the solution but was also closely correlated to the morphological changes in its hydrolysates and the competitive adsorption with other cations. Therefore, the adsorption properties of Taiwan Zhi-Shin bentonite on Sr^2+^ were shown to be more suitable under alkaline conditions (pH > 9).

#### 2.3.3. Effect of Ionic Strength on Adsorption

**Figure 6:**
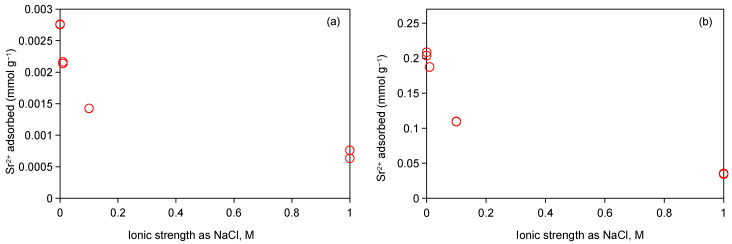
0.1 mM-Sr adsorption (**a**) and 10 mM-Sr (**b**) adsorption on Taiwan Zhi-Shin bentonite as affected by solution ionic strength.

The competitive cation exchange mechanism governing Sr^2+^ adsorption on Taiwan Zhi-Shin bentonite was further validated through ionic strength experiments. Studies have shown that due to its valence layer being full and its inability to form strong coordination bonds with surface sites, Sr^2+^ was mainly adsorbed on the surface at the clay-water interface in the form of a mononuclear exo-spherical complex, which is weakly bound, easily replaced by competing cations, and displays high migration [55]. As depicted in Figure 6, incremental increases in NaCl concentration (0.01 M, 0.1 M, and 1 M) induced a marked reduction in Sr^2+^ adsorption capacity. At 0.1 mM-Sr, adsorption decreased from 0.0027 mmol g^−1^ to 0.0008 mmol g^−1^ (Figure 6a), while at 10 mM-Sr, the adsorption capacity declined from 0.21 mmol g^−1^ to 0.04 mmol g^−1^ (Figure 6b). This indicates that the competitive effect of cation exchange was enhanced with the increase in sodium ion concentration, which leads to the decrease in Sr^2+^ adsorption. In addition, compared with the low ionic strength (0–0.1 M NaCl), the adsorption of Sr^2+^ decreased rapidly. This result is consistent with the experimental results of Sr^2+^ adsorption on other clay minerals [55], which further verifies that cation exchange is the main mechanism of Sr^2+^ adsorption.

#### 2.3.4. Effect of Temperature on Sr^2+^ Adsorption

**Figure 7:**
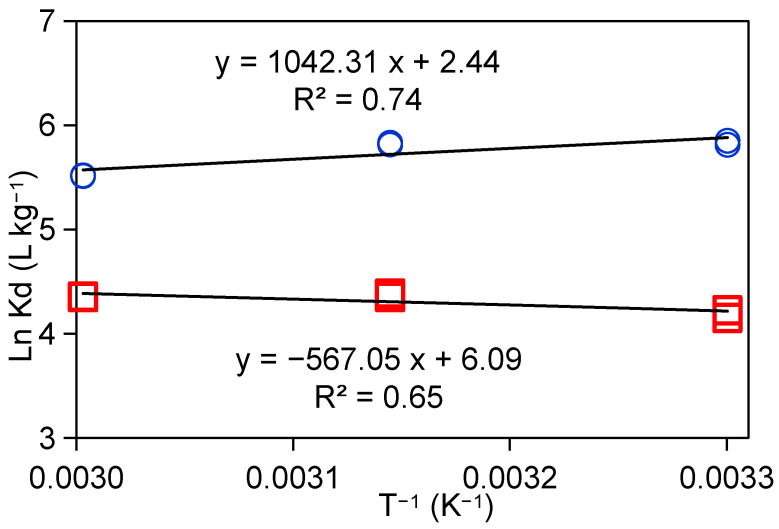
The 0.1 mM-Sr adsorption and 10 mM-Sr adsorption on Taiwan Zhi-Shin bentonite as affected by equilibrium temperature.

Under 0.1 mM-Sr and 10 mM-Sr, the adsorption properties of Taiwan Zhi-Shin bentonite on Sr^2+^ at 30 °C, 45 °C, and 60 °C were investigated, respectively, and the results are shown in Figure 7. By plotting LnKd against 1/T (van’t Hoff plot), the relevant thermodynamic parameters for the adsorption of Sr^2+^ by Taiwan Zhi-Shin bentonite can be calculated [56]. The calculated results of thermodynamic parameters are shown in Table 2. Under the condition of 0.1 mM-Sr, the calculated enthalpy change is less than zero (ΔH < 0). The adsorption of Sr^2+^ by Taiwan Zhi-Shin bentonite is an exothermic reaction, and high temperature is not conducive to adsorption, which is consistent with the slightly decreasing trend of adsorption with increasing temperature in the experiment. It is worth noting that the experimental results are consistent with the adsorption results of Sr^2+^ on dolomite powder (C_i_ = 10 mol L^−1^), PAN/zeolite (C_i_ = 150 µg mL^−1^), attapulgite (C_i_ = 40 µg mL^−1^), and Al_2_O_3_ (C_i_ = 50 µg mL^−1^) [57,58,59,60]. These results occur because when the concentration of Sr^2+^ in the system is low, there are many active sites on the surface of the adsorbent, and the heat required for Sr^2+^ to be adsorbed on the surface of Taiwan Zhi-Shin bentonite is relatively small, so the process shows a trend of exothermic heat at low concentration. In addition, the negative value of the free energy variation (ΔG = −6.1 kJ mol^−1^) further demonstrated the spontaneity of the adsorption process. The negative value of entropy change (ΔS = −0.01 kJ mol^−1^ K^−1^) indicated that the system disorder was reduced during adsorption [61], which may be due to the fact that Sr^2+^ is fixed in a specific position on the surface of Taiwan Zhi-Shin bentonite, restricting its free movement.

Under the adsorption system of 10 mM-Sr, the adsorption of Sr^2+^ by Taiwan Zhi-Shin bentonite increased with the increase in temperature, which was opposite to the trend of adsorption at low concentration, indicating that the adsorption process of Sr^2+^ at high concentration was an endothermic reaction (ΔH = +4.71 kJ mol^−1^). The increase in temperature was conducive to the adsorption, which is consistent with the experimental results. The observation in this scenario can be explained as follows: as the concentration of Sr^2+^ increases, more Sr^2+^ will be adsorbed on the adsorbent, the adsorption of the active site will be relatively reduced, and the surface of the adsorbent will become more crowded. Previous studies have shown that a pyrochlore-type material is a material with a defect (vacancy) structure. Perhaps due to steric hindrance, the adsorption of Sr^2+^ requires a stronger chemical bond force, and the corresponding thermal effect in the adsorption process will increase [62]. The positive value of entropy change (ΔS = +0.02 kJ mol^−1^ K^−1^) indicates that the system disorder increases during the adsorption process, which may be due to the fact that more Sr^2+^ is adsorbed to the surface of Taiwan Zhi-Shin bentonite at high concentration, and the mobility of Sr^2+^ ions and surrounding water molecules increases due to ionic dehydration [63].

### 2.4. X-Ray Diffraction

**Figure 8:**
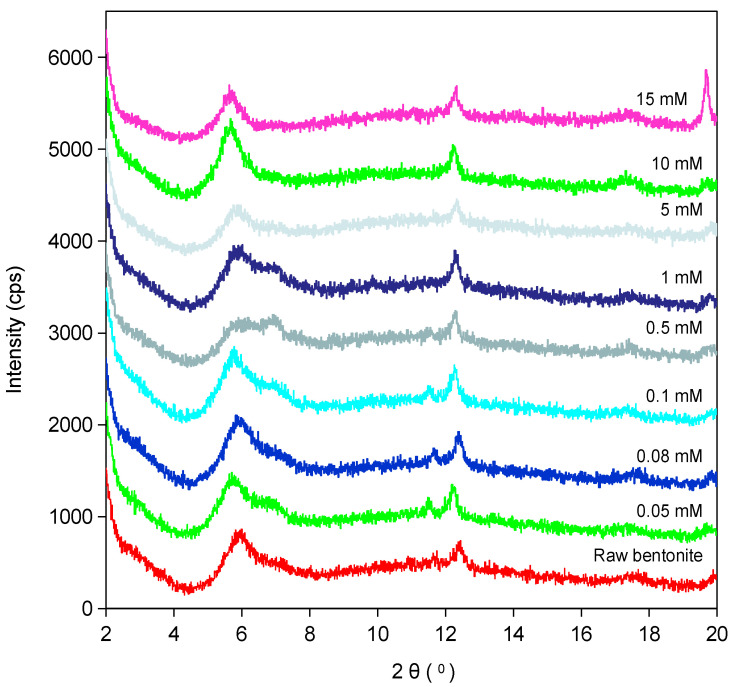
XRD pattern of raw Taiwan Zhi-Shin bentonite and Sr-adsorbed Taiwan Zhi-Shin bentonite under different adsorbed amounts.

Taiwan Zhi-Shin bentonite is mainly composed of montmorillonite and varying amounts of other minerals, such as quartz (SiO_2_), kaolinite, and pyrite [49]. As shown in Figure 8, Sr^2+^, by substituting interlayer Ca^2+^ and carrying a double-layer hydration molecule (2 W), is inserted into the interlayer of montmorillonite [64,65], causing its (001) crystal plane [66,67] base spacing (d_001_) to extend from 14.71 Å to 15.6 Å (pH = 9). This is consistent with the heterogeneous adsorption properties revealed by the Freundlich isothermal model (*n* > 1.2). The (001) crystal plane (2θ ≈ 12°) distance of kaolinite extends slightly from 7.1 Å to 7.23 Å [68,69], confirming the auxiliary contribution of the coordination of surface hydroxyl groups with Sr^2+^ to adsorption, forming a synergistic effect under the mineral complex system [70,71]. In addition, there was no indication that large quantities of other minerals are present in the 2θ range of 5–20° (Figure 8).

The dynamic properties of the adsorption process are regulated by the Sr^2+^ concentration in conjunction with the pH of the solution. In the low concentration range (0.05–5 mM), the uneven distribution of layer charges led to an alternating arrangement of Sr^2+^ interstratification (2 W hydrated, d_001_ ≈ 15.5 Å) with an uninterpolated region (1 W hydrated, d_001_ ≈ 12.5 Å) (Figure 8). There was spatial heterogeneity present, reflecting the competitive exchange of Sr^2+^ and Ca^2+^ [72]. This change can be attributed to the change in the hydration state of the exchanged and adsorbed cation Sr^2+^ and the coexistence of interstratified structures with varying hydration states, resulting in dynamic adjustments of the interlayer structure [73]. As the concentration increases to 10–15 mM, adsorption approaches saturation, the interlayer disappears, and the interlayer expands uniformly to 15.6 Å, indicating full occupation of the interlayer site by Sr^2+^. This phenomenon is consistent with the expansion behavior of montmorillonite reported in previous literature [74,75].

**Figure 9:**
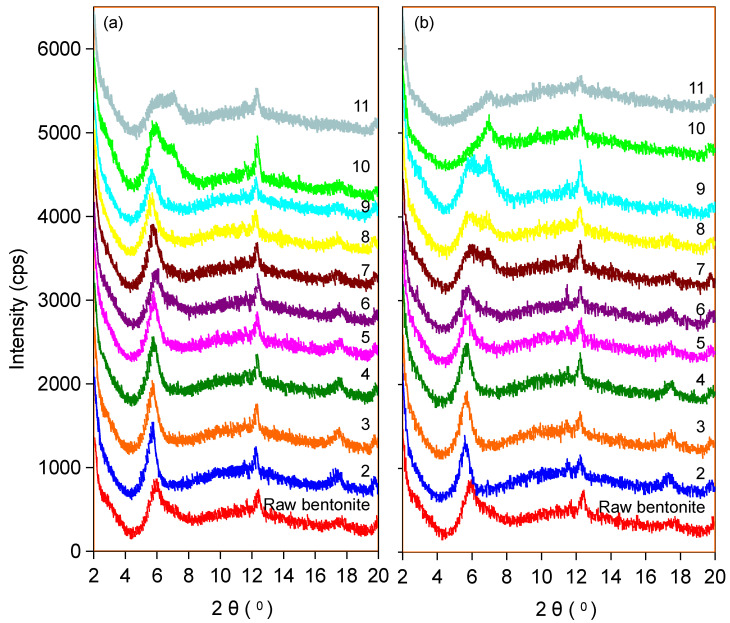
XRD pattern of 0.1 mM-Sr adsorbed on Taiwan Zhi-Shin bentonite (**a**) and raw bentonite (**b**) under different pHs.

The effect of pH on the adsorption system of 0.1 mM-Sr is manifested in two-stage differentiation (Figure 9a): under acidic conditions (pH < 7), H^+^ competition leads to partial desorption of Ca^2+^ and Sr^2+^ insertion into the interlayer with restricted hydration (d_001_ ≈ 15.0 Å). However, under alkaline conditions (pH > 7), Ca^2+^ retention stabilizes, and Sr^2+^ deep intercalation efficiency increases (d_001_ ≈ 15.6 Å). Under an extremely alkaline environment (pH > 10), the interlayer hydroxylation leads to the reconfiguration of hydration balance, and the interlayer phenomenon reappears, suggesting the reorganization of the interlayer chemical microenvironment after adsorption saturation. This is attributed to the fact that there was little desorption of Ca^2+^ after pH > 7, while adsorption saturation was achieved at pH > 10, as indicated by studies of pH absorption edge effects and cation resolution (Figure 4a and Figure 5a).

**Figure 10:**
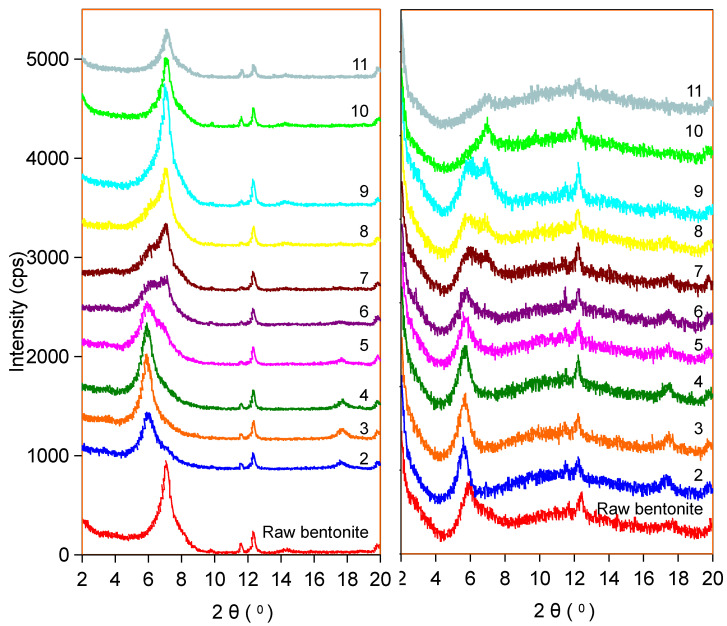
XRD pattern of 10 mM-Sr adsorbed on Taiwan Zhi-Shin bentonite (**a**) and raw bentonite (**b**) under different pHs.

The comparative study with raw bentonite before adsorption showed that Taiwan Zhi-Shin bentonite displayed higher stability (pH > 7 without interlayer) under the 0.1 mM-Sr system, which may be due to the cushioning effect of kaolinite on the interlayer charge and the synergistic effect of minerals. However, the raw bentonite generally exhibited interlayer shrinkage under the same conditions (d_001_ recovered to 12.0 Å when pH > 10), highlighting the structural advantages of Taiwan Zhi-Shin bentonite (Figure 9b and Figure 10b). Under the 10 mM-Sr system, the adsorption capacity of Taiwan Zhi-Shin bentonite increased abruptly to 0.23 mmol g^−1^ when at pH > 9 (Figure 4b), resulting in the expansion of d_001_ spacing from 12.0 Å to 15.5 Å and the emergence of the interlayer at pH 5, and the interlayer homogenization was gradually completed after pH > 8 (Figure 10a). Combined with the evidence of precipitation of no Sr(OH)_2_, it was further confirmed that the adsorption mechanism is dominated by ion exchange rather than surface precipitation.

Thus, the adsorption of Sr^2+^ on Taiwan Zhi-Shin bentonite is based on interlayer ion exchange in the form of montmorillonite (Sr^2+^ replaces Ca^2+^ and carries hydrated molecules to vacate the layer intervals), supplemented by a synergistic effect of surface adsorption in kaolinite. The adsorption dynamics were regulated by concentration and pH, with intercalations coexisting at low concentrations and high concentrations or alkaline conditions towards uniform intercalation. Its multi-mechanism coordination and interlayer dynamic adjustment characteristics make it have high adsorption potential in the remediation of radioactive Sr^2+^ contamination.

### 2.5. Molecular Simulation

**Figure 11:**
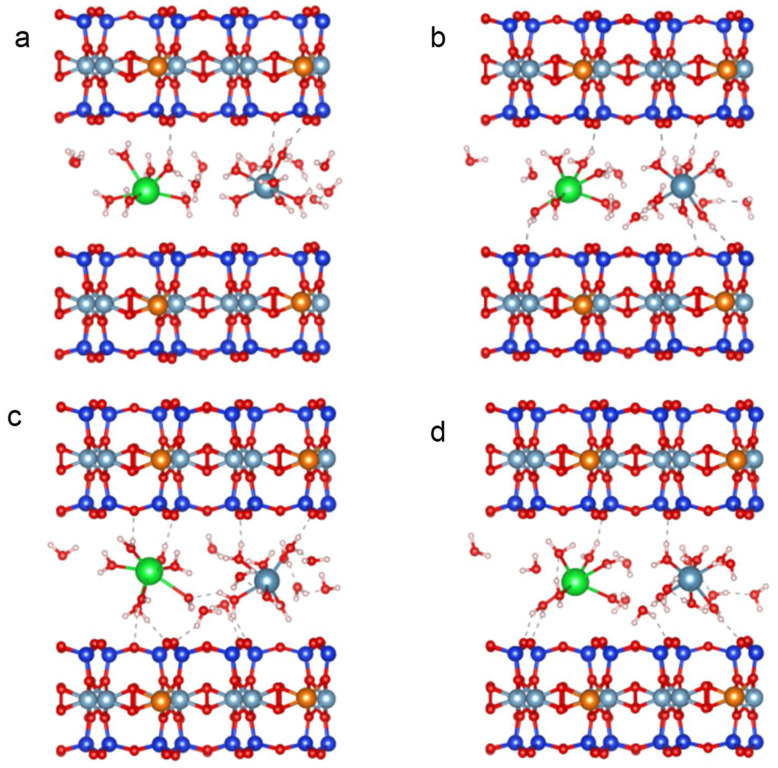
Hydrogen bonds and water “bridges” are formed between Sr^2+^ and the bentonite surface. (**a**) The initial configuration of Sr^2+^ within the Ca^2+^ bentonite layer; (**b**) a snapshot of the AIMD simulation showing the formation of hydrogen bonds between water on the hydrated surface and oxygen atoms on the bentonite surface; (**c**) snapshots of AIMD simulations, the formation of hydrogen bonds between hydrated ions and bentonite surfaces, and the formation of water “bridges” connecting Sr^2+^ and Ca^2+^ surfaces; (**d**) a snapshot of the AIMD simulation, the formation of a water “bridge” connecting Ca^2+^ to the bentonite surface.

Through AIMD simulation, the adsorption mechanism of Sr^2+^ on the bentonite surface was studied, with special attention to the molecular scale interaction. As can be seen from Figure 11a, on the surface of Ca-bentonite, hydrated Ca^2+^ and Sr^2+^ formed hydrogen bonds with oxygen atoms on the surface of bentonite through surface water. As the simulation progresses, Figure 11b shows that the number of hydrogen bonds formed by Ca^2+^ and Sr^2+^ with the bentonite surface increases. Surprisingly, we did not find a directly related adsorption mechanism of Sr^2+^ on bentonite, given that Sr^2+^ has similar properties to calcium [2], Moreover, it has been reported that the adsorption of Ca^2+^ on bentonite is achieved through hydrogen bonding [76], so it can be inferred that Sr^2+^ is mainly bonded with oxygen atoms on the surface of bentonite through hydrogen bonding by binding water.

To quantify the interaction of Sr^2+^ on bentonite, adsorption energy calculations were performed on a representative simulation framework exhibiting hydrogen bonds. According to the energy analysis, the relative adsorption energy of Sr^2+^ on Ca-bentonite is −16.08 eV. For comparison, the adsorption energy of Ca^2+^ on a molybdenite surface is −10.14 eV [77]. The adsorption energies of Cr^2+^ on asphaltene-pyrrole and asphaltene-thiophene surfaces are −5.89 eV and −3.08 eV, respectively [78]. The adsorption energy of Ca^2+^ on the bentonite surface is −15.55 eV [79]. These data indicate that Sr^2+^ has a strong interaction on Ca-bentonite.

Figure 11c shows that hydrated Ca^2+^ and Sr^2+^ are connected by surface water through a bond called a bridge. These bridges are able to increase the adsorption of Sr^2+^ between bentonite layers. This suggests that hydrogen bonds and water bridges coexist during the adsorption of Sr^2+^, and the two mechanisms synergistically contribute to the adsorption of Sr^2+^ to the bentonite surface. Figure 11d shows that there also is a water bridge between Ca^2+^ and bentonite. Due to the short time of molecular dynamic simulation, we were not able to determine whether hydrogen bonds are formed between Sr^2+^ and bentonite, but given that Sr^2+^ has similar properties to calcium [2], it is highly likely that water bridge bonds also exist between Sr^2+^ and bentonite.

In addition, no other adsorption mechanism was observed through this simulation, so a DFT energy analysis was conducted, and it was determined that the energy of Ca^2+^ on bentonite is −15.55 eV, which is consistent with the results of Xing et al. [79]. Using the same method, it could be shown that the energy of Sr^2+^ on bentonite is −15.30 eV. The results show that the adsorption energy of Ca^2+^ was slightly greater than that of Sr^2+^ [80]. In addition, through the molecular simulation comparison of Na-bentonite and Ca-bentonite on Sr^2+^, it was determined that Na-bentonite displayed the same mechanism on Sr^2+^, mainly hydrogen bonding and water bridging (Figure S1). According to DFT energy analysis, the adsorption energy of Sr^2+^ on bentonite (15.27 eV) was twice that of Na^+^ (7.1 eV), so the adsorption of Sr^2+^ on Na-bentonite involves ion exchange.

Therefore, according to the results of molecular dynamics simulation, it can be inferred that the adsorption mechanism of Sr^2+^ on Ca-bentonite mainly involves hydrogen bonding and a water bridge. During the adsorption process, the water molecules between the bentonite layers play a synergistic role, which enhances the interaction between Sr^2+^ and the Taiwan Zhi-Shin bentonite surface.

## 3. Discussion

The adsorption mechanism of Sr^2+^ by Taiwan Zhi-Shin bentonite exhibited multi-scale synergistic characteristics. The slope of 0.84 between the adsorption capacity of Sr^2+^ and the desorption of cations confirms that cation exchange was the primary mechanism for Sr^2+^ adsorption on Taiwan Zhi-Shin bentonite, with Ca^2+^ (accounting for 60.4% of the desorbed cations) being the main exchanged ion, while Na^+^ desorption was relatively low. However, the total desorbed cation quantity was less than the adsorbed Sr^2+^ concentration, indicating that surface complexation may have also played a significant role alongside cation exchange. This dual-mechanism synergy is further corroborated by XRD data: as Sr^2+^ concentration increases, the expansion of the (001) crystal plane basal spacing (d_001_) from 14.71 Å to 15.6 Å demonstrates the existence of interlayer adsorption sites in Taiwan Zhi-Shin bentonite, confirming Sr^2+^ intercalation into the montmorillonite interlayers. The selective replacement of Ca^2+^ by Sr^2+^ in the montmorillonite interlayer space, attributed to the Sr^2+^ larger ionic radius and its bilayer hydration molecules (adsorption energy: −15.3 eV), leads to ion exchange-driven interlayer expansion and potential local structural reorganization caused by surface complexation, resulting in interstratification phenomena [73,81]. This process aligns with the high adsorption capacity (*n* > 1.2) revealed by the Freundlich isotherm model, indicating multilayer heterogeneity and energetic heterogeneity of surface adsorption sites [43]. Additionally, the presence of minor kaolinite in Taiwan Zhi-Shin bentonite further enhanced Sr^2+^ adsorption through synergistic effects.

The adsorption behavior of Sr^2+^ by Taiwan Zhi-Shin bentonite exhibited significant concentration dependence. At low concentrations, the process was exothermic and entropy-decreasing, dominated by spontaneous adsorption driven by electrostatic interactions and hydration effects. In contrast, at high concentrations, the adsorption transitioned to an endothermic and entropy-increasing mechanism characterized by strong chemical bonding (outer-sphere complexation) [52], demonstrating Taiwan Zhi-Shin bentonite’s broad adaptability in complex aqueous environments. The enthalpy change (ΔH) correlated with concentration gradients, active site density, and chemical bond strength, while the entropy variation (ΔS) likely stemmed from Sr^2+^ dehydration and enhanced mobility of interlayer water molecules [63], further validating mechanistic differences across concentration regimes. AIMD simulations of Sr^2+^-loaded adsorption at the molecular scale revealed that Sr^2+^ and Ca^2+^ form hydrogen-bond networks via interfacial water molecules bridging oxygen atoms on Taiwan Zhi-Shin bentonite surfaces. The relative adsorption energy of Sr^2+^ (−15.3 eV) exceeded that of Ca^2+^ (−12.1 eV), confirming the preferential binding of Sr^2+^ through stronger interactions. Moreover, Sr^2+^ and Ca^2+^ are interconnected by water-bridge structures, which enhance the ordered arrangement of interlayer water molecules, stabilize Sr^2+^ adsorption sites through coordinated hydration shells, and enable high-efficiency adsorption. This dual mechanism—thermodynamic adaptability and molecular-level structural optimization—explains Taiwan Zhi-Shin bentonite’s exceptional performance in Sr^2+^ sequestration under varying environmental conditions.

The adsorption kinetics of Sr^2+^ on Taiwan Zhi-Shin bentonite were shown to be significantly regulated by Sr^2+^ concentration and solution pH. The adsorption process followed a pseudo-second-order model (R^2^ = 1.000), achieving rapid equilibrium within 15 min due to the high accessibility of montmorillonite interlayer cation exchange sites [82]. The XRD results confirmed that the hydrated ions of Sr were inserted into the interlayers of Taiwan Zhi-Shin bentonite during adsorption, while pH-dependent studies further revealed the dynamic heterogeneity in adsorption behavior: under low Sr^2+^ concentrations (0.1 mM), acidic conditions (pH < 7) induced partial Ca^2+^ desorption via H⁺ competition, allowing Sr^2+^ insertion in a restricted hydration state (d_001_ ≈ 15.0 Å); alkaline conditions (pH > 7) enhanced Sr^2+^ deep intercalation efficiency (d_001_ ≈ 15.6 Å) by stabilizing Ca^2+^ retention. Under high-concentration systems (10 mM), the adsorption capacity increased markedly with pH (reaching 0.23 mmol g^−1^ at pH > 9), accompanied by d_001_ expansion from 12.0 Å to 15.5 Å, indicating complete Sr^2+^ occupancy of interlayer sites and suppression of interstratification. Additionally, elevated Sr(OH)⁺ concentrations at pH > 9 enhanced adsorption via electrostatic attraction, while weakened Ca^2+^ competitive displacement due to precipitation formation in alkaline conditions further optimized efficiency. The synergistic interplay of cation exchange, surface complexation, electrostatic interactions, water-bridge assistance, and dynamic interlayer adjustments enables Taiwan Zhi-Shin bentonite to achieve high-efficiency Sr^2+^ removal (>95% within 5 min) across 0.1–10 mM concentrations, with superior ion interference resistance compared to synthetic materials, providing a natural mineral-based solution for rapid Sr^2+^ treatment in nuclear wastewater.

## 4. Materials and Methods

### 4.1. Materials

#### 4.1.1. Preparation of Simulated Nuclear Waste Liquid

Sr is an alkaline earth metal with a molecular weight of 87.62 g mol^−1^, a density of 2.60 g cm^−3^, a boiling point of 1384 °C, and a melting point of 769 °C [83]. It is an active positively charged metal ion that is easily oxidized to the stable and colorless Sr^2+^, with chemical properties similar to calcium and barium [1]. In this study, Sr(NO_3_)_2_ was used to prepare a simulated nuclear waste solution containing Sr^2+^ with a pH of 7 and a mass concentration of 0.5 mM.

#### 4.1.2. Taiwan Zhi-Shin Bentonite

Taiwan Zhi-Shin bentonite is produced by Zhi Hing Mining Co., Ltd. (Yunlin, Taiwai), which is located in the Duluan Mountain layer of Zhangyuan Village, Changbin Township, Taitung County. Taiwan Zhi-Shin bentonite was adopted for these experiments. The sample was lumpy and was pulverized into powder for use without further purification. Through our literature investigation, the CEC of Taiwan Zhi-Shin bentonite was determined to be 59, 60.1~72.0, 86 meq 100 g^−1^. This may be due to differences in sampling time and location. The Taiwan Zhi-Shin bentonite in this study is 80~86 meq 100 g^−1^ after three measurements with the ammonium saturation method, and the main exchange cation was shown to be Ca^2+^ [84]. The experimental results show that the PZC is 9.2. The chemical composition of the Taiwan Zhi-Shin bentonite is given in Table S4.

### 4.2. Experimental Methods

#### 4.2.1. Batch Experiment

An amount of 1 g of Taiwan Zhi-Shin bentonite was added to several centrifuge tubes containing 30 mL of simulated nuclear waste liquid (S/L = 33.3 g L^−1^), and then the centrifuge tubes were placed in an oscillator (150 r min^−1^) to carry out oscillatory adsorption experiments at room temperature. In the dynamic experiment, the centrifugal tubes were removed successively after 1, 2, 5, and 10 min and 0.25, 0.5, 1, 2, 4, 8, 16, and 24 h of oscillations. In the other batches of experiments, the tubes were removed after 24 h. The tube was centrifuged at 8000 r min^−1^ for 5 min, and the supernatant was collected in plastic bottles.

In the adsorption isotherm experiment for Sr^2+^, the initial solution concentration was set at 0.05, 0.08, 0.1, 0.5, 1, 5, 10, and 15 mmol L^−1^, and the solid-liquid ratio was 33.3 g L^−1^. The oscillatory adsorption experiment was carried out in an oscillator (150 r min^−1^) at room temperature. After 24 h, the centrifuge tubes were removed and centrifuged at 8000 r min^−1^ for 5 min, and the supernatant was collected in the spare plastic bottle.

In the other group of experiments, the initial concentration of Sr^2+^ was about 10 mmol L^−1^, and the remaining experimental conditions were the same. An amount of 30 mL of Sr^2+^ solution with a concentration of 0.1 mmol L^−1^ was added to the plastic centrifuge tube, and 1 g of Taiwan Zhi-Shin bentonite was added to each centrifuge tube, which was put into the shaking table. The temperature was maintained at 25 °C, and the rotating speed was set at 150 rpm. The pH was adjusted to between 2 and 11 using diluted hydrochloric acid or sodium hydroxide solution. After oscillating for 1, 2, 5, and 10 min and 0.25, 0.5, 1, 2, 4, 8, 16, and 24 h, the centrifuge tubes were removed successively, the supernatant was centrifuged on the centrifuge (8000 r min^−1^) for 5 min, and the supernatant was collected in the spare plastic bottle.

#### 4.2.2. Analytical Methods

Sr^2+^ concentrations were measured by inductively coupled plasma optical emission spectroscopy (Vista-MPX, Agilent Technologies Inc., Jinan, China). Samples were diluted and acidified in 1% HNO_3_ solution. The analysis device adopted an MPX CCD detector and RF generator and applied direct serial coupling technology. The V-groove atomizer and the two-pass Sturman–Masters atomizer were introduced, and the peristaltic pump was also set up. In addition, the spectrometer operated in a transient signal acquisition mode [85].

XRD analysis was performed on a Rigaku Ultima IV (Nanjing, China) diffractometer equipped with a D/teX Ultra (CuKα radiation) detector using X-ray diffractometer (XRD) at a scanning speed of 2° min^−1^ and a step increment of 0.01 in the range of 2θ 2–65°. The adsorption mechanism of Sr^2+^ on the Taiwan Zhi-Shin bentonite surface was analyzed.

#### 4.2.3. Molecular Simulation

The structure of bentonite consists of two layers of Si-O tetrahedrons and an Al-O octahedron sandwiched between them [31]. In order to improve computational efficiency, we constructed a cell model with a size of 2a × 1b × 1c (17.96 Å × 10.36 Å × 39.00 Å) supercell. The distance between bentonite layers measured by experiment is 14.7 Å. In the lattice, four Al^3+^ are replaced by Mg^2+^ and Ca^2+^ ions are placed in the interlayer space to maintain charge balance. In addition, 22 water molecules were introduced into the interlayer region, of which six formed a hydrated shell around the Ca^2+^ ion and another six formed a hydrated shell around the Sr^2+^ [86,87].

The present study utilized the Quickstep module of the CP2K package [88] to perform ab initio molecular dynamics (AIMD) simulations of Sr^2+^ ions in Ca-bentonite based on density functional theory (DFT) [89]. The Perdew–Burke–Ernzerhof (PBE) [90] exchange correlation function combined with Grimme’s DFT-D3 correction [91] was used to describe the dispersion effect. The Goedeck–Teter–Hutter (GTH) [92] pseudopotential was used to model the interaction between the core electrons and the nucleus. Due to the large number of electrons involved, we used GTH bi-zeta valence polarization (DZVP) Gaussian basis combined with plane wave basis function and set the energy cutoff value to 500 Ry. The orbital transformation (OT) method [93] was used to optimize the wave function, and the strict convergence threshold was set to 1 × 10^−6^ atomic units. In the simulation of molecular dynamics, we used a canonical (NVT) [94] ensemble with a temperature set at 330 K and a Nosé–Hoover thermostat [95] to maintain the temperature. The total simulation time was approximately 856 fs with a time step of 0.5 fs to ensure a thorough analysis.

## 5. Conclusions

The absorption of Taiwan Zhi-Shin bentonite, which is dominated by calcium-based montmorillonite, is mainly based on ion exchange and surface complexation of montmorillonite crystals at low pH and Sr^2+^ concentration. In highly concentrated Sr^2+^ solutions, the adsorption is mainly based on ion exchange between the clay-crystal cations and the external medium Sr^2+^. At pH > 10 electrostatic interactions were also shown to be involved, and the hydration ions of Sr^2+^ and Ca^2+^ may form a water bridge co-existing between mineral layers. Minerals like bentonite are abundant in output. Moreover, compared with other adsorbents used in similar processes, Zhi-Shin bentonite has the characteristics of simple operation and low cost.

However, the expansion performance of Taiwan Zhi-Shin bentonite is not as good as that of sodium bentonite abroad, so it is usually necessary to improve the expansion characteristics through physical, chemical, heat treatment, and ion exchange methods to improve its competitiveness as a buffer material.

## Figures and Tables

**Table 1:** Estimated isothermal model parameters for Sr^2+^ absorption onto Taiwan Zhi-Shin bentonite.

Adsorbents	Langmuir Model			Freundlich Model		
	q_e_ exp (mmol g^−1^)	q_max_ (mmol g^−1^)	R^2^	K_F_	*n*	R^2^
Zhi-Shin bentonite	0.28	0.41	0.4945	0.112	1.196	0.96

q_e_ exp: actual adsorption value; q_max_: theoretical maximum adsorption value; R^2^: correlation coefficient; K_F_ and *n*: Freundlich constant.

**Table 2:** Thermodynamic parameters of Sr^2+^ uptake on Taiwan Zhi-Shin bentonite under different temperatures.

C_i_	pH	LnKd (L Kg^−1^)			ΔG° (kJ mol^−1^)			ΔH° (kJ mol^−1^)	ΔS° (KJ mol^−1^ K^−1^)
		303	318 K	333 K	303 K	318 K	333 K		
0.1 mM-Sr	9	2.43086	2.42254	2.11603	−0.62492	−6.1295	−6.0099	−8.67	−0.01
10 mM-Sr	9	0.78774	0.96798	0.9534	−2.06	−2.73	−2.73	4.71	0.02

LnKd: logarithm of the retention factor; ΔG°: Gibbs free surface energy change; ΔH°: change in standard enthalpy; ΔS°: change in standard entropy.

## Data Availability

Data are contained within the article and Supplementary Materials.

## Supplementary Materials

The following supporting information can be downloaded at https://www.mdpi.com/article/10.3390/ijms26115298/s1. References [96,97,98,99,100,101] are cited in the Supplementary Materials.
