# Supplementary material for: Adsorption of Sr2+ from Synthetic Waste Effluents Using Taiwan Zhi-Shin Bentonite
Source: Int J Mol Sci. 2025 May 30;26(11):5298. doi: 10.3390/ijms26115298 (PMC12155143; doi:10.3390/ijms26115298)
Supplement: Supplementary file 1 [file ijms-26-05298-s001.zip › ijms-3619397-supplementary.pdf]

Adsorption of Sr^{2+} from synthetic waste effluents using Taiwan Zhi-Shin bentonite

Yi-Hui Lin [1,†], Yuhan Li [2,†], Yating Yang [2,*] and Po-Hsiang Chang [2,*]

1 Department of Geography, Hanshan Normal University, Chaozhou 521041, China; 20190031@hstc.edu.cn(Y.L.)

2 College of Resources and Environment, Fujian Agriculture and Forestry University, Fuzhou 350002, China; yuhan200203@163.com (Y.L.);

† The two authors contributed equally to the work.

* Correspondence: 12308059030@fafu.edu.cn (Y.Y.); phchang@fafu.edu.cn (P.-H.C.)

Table S1 The advantages and disadvantages of the method of removing radioactive ions

Method	Advantage	Disadvantage	References
Ionic Exchange	Simple operation	Radiation resistance, heat resistance and chemical resistance are poor, the cost is high, and the regeneration is difficult	[1-3]
Chemical precipitation	The process is simple, the cost is low and the application range is wide	After use, it is difficult to separate solid and liquid, the amount of sludge is large and there is secondary pollution	[4]
Membrane separation	Energy saving, environmental protection, high efficiency, economy, easy to control	Performance is easy to reduce, short life	[5]
Evaporation and concentration	High purification coefficient, strong flexibility, wide application range, can be combined with a variety of technologies, no secondary pollution	High energy consumption, scaling, explosion and other conditions occur, easy to corrode	[6[7]
Biotechnology	Environmental protection, high efficiency, mild, low cost and no secondary pollution	There are requirements for radiation intensity	[89]
Photocatalysis	Low cost, high safety, high efficiency, no secondary pollution	The separation efficiency is low and the utilization rate of sunlight is low	[10]
Adsorption	Simple operation, multi-functional chemical stability, easy to modify<br>Montmorillonite has large reserves, low price, strong expansibility and wide application range	Although some adsorption materials (MOF, graphene oxide and resins, etc.) have good adsorption performance, they have high cost, complex adsorption technology requirements, and are not used in actual environmental applications.	[11-15]

Table S2 Comparison of maximum Sr^{2+} adsorption capacity of various adsorbents.

	adsorbent	Ci (mg L^{-1})	Maximum adsorption capacity (mg g^{-1})	Solution pH	Best fitted isothermal model	Reference
Rare earth oxalate framework	Eu-ox	13.2–703	92.17		Langmuir-Freundlich model	[16]
	Y-ox	13.2–703	85.23		Langmuir-Freundlich model	
Hydrogel	Thiol-rich and ion-imprinted alginate hydrogel	100-500	151.7		dual-site Langmuir model	[17]
Resin	Dowex-HCR-S/S	100-500	400		Langmuir	[18]
	Sulfonylcalix [4] arene-loaded XAD-7 Resin	10-70	28.87		Langmuir	[19]
	Dowex 50W-X Resins	20-100	134.62		Redlich-Peterson model parameters (R-P isotherm)	[13]
Mineral	basic zeolites	1-50	56.2		Langmuir	[20]
	Na- bentonite	25-100	45.7-47.6		Langmuir	[21]
	graphene oxide	20-215	131.41		Langmuir	[14]
	Anionic layered CP material（SZ-4）	1-400	117.9		Langmuir	[22]
MOF	MOF/KNiFC	50-3500	114	5.5	Langmuir	[15]
	MOF/Fe_3O_4/KNiFC	50-3500	94	5.5	langmuir	
Synthetic material	Synthesized antimony silicate	2-10	43.9	4	Freundlich model	[23]

Ci: initial concentration (mg L^{-1})

Table S3 The relevant parameters of the kinetic model of adsorption of Sr^{2+} by various materials were studied.

	Adsorbent	C_i (mg L^{-1})	Adsorbent mass (g)	Solution (mL)	pH	Adsorbing capacity (mg g^{-1})	Equilibrium time	Reference
Rare earth oxalate framework	Eu-ox	2.5	0.05	50		2.49	3min	[16]
Hydrogel	Thiol-rich and ion-imprinted alginate hydrogel	100	0.05	50	7	46.9	30min	[17]
Resins	Dowex-HCR-S/S	100	0.01	15		154.56	3h	[18]
	Sulfonylcalix [4] arene-loaded XAD-7 Resin	20	0.01	10	6	-	7h	[19]
	Dowex 50W-X Resins	40	0.01	50		13.89	1h	[13]
Mineral	basic zeolites	50	0.05	50		50	6h	[20]
	Na- bentonite	50	0.02	50		35-43	11min-14min	[21]
	graphene oxide	100	0.02	100	5	66.05	18min	[14]
	Anionic layered CP material (SZ-4)	10	0.02	10	4	92%	18min	[22]
MOF	MOF/KNiFC	1000	0.05	-	5	114.9	45min	[15]
	MOF/Fe_3O_4/KNiFC	1000	0.05	-	5	106.38	45min	
Synthetic material	Synthesized antimony silicate	4	0.01	40		14.5	3h	[23]

C_i: initial concentration (mg L^{-1})

Table S4 Chemical composition of Taiwan Zhi-Shin bentonite[24]

Composition	SiO_2	Al_2O_3	Fe_2O_3	CaO	Na_2O	K_2O
ZS Bentonite (Taiwan)	50.87	15.54	5.93	2.83	1.21	1.41
Composition	MnO	MgO	TiO_2	P_2O_5	LOI	Total
ZS Bentonite (Taiwan)	0.1	2.11	0.38	0.07	14.72	97.17

LOI loss in ignition

（1）Langmuir adsorption isotherm

In 1918, Langmuir derived the following Langmuir adsorption isotherm formula according to the adsorption equilibrium state reached when the condensation rate of gas to the solid surface of the adsorbent and the evaporation rate from the surface were equal[25]：

$$C_S = \frac{K_L S_m C_L}{1+K_L C_L} \qquad (S1)$$

Linear expression is：

$$\frac{C_L}{C_S} = \frac{1}{K_L S_m} + \frac{C_L}{S_m} \qquad (S2)$$

Among them, C_L and C_S are the concentration of Sr^{2+} at adsorption equilibrium (mmol L^{-1}) and the adsorption amount of Sr^{2+} by Taiwan bentonite (meq g^{-1})，S_m is the maximum adsorption capacity of Sr^{2+} by Taiwan bentonite , and K_L is the equilibrium constant related to adsorption capacity. The establishment of Langmuir adsorption theory is mainly based on the following ideal assumptions: 1) there are uniformly distributed adsorption sites on the solid surface, and each adsorption site can only adsorb one atom or molecule, that is, single-molecular layer adsorption; 2) The adsorption between the adsorbent and the adsorbent is similar to a reversible chemical reaction, and there is no interaction between the molecules of the adsorbent. This theory is effective for the single molecular layer adsorption on the surface of the homogeneous adsorbent. However, due to the complexity of the actual adsorption process, the adsorption behavior can not fully conform to the reversible reaction, and there are generally multi-molecular layer adsorption phenomena, so there are certain limitations in practical application.

(2) Freundlich adsorption isotherm

The Freundlich isotherm equation was originally obtained by experiment and is an empirical equation. This theory takes into account the uneven distribution of adsorbent surface adsorption energy due to pore size distribution and load functional groups, and the different adsorption energy of each layer in multilayer adsorption. It assumes that the adsorption heat decreases exponentially with the increase of adsorption degree, and is suitable for many cases of physical adsorption and chemical adsorption[26]. The relation is：

$$q_e = K_F {C_e}^{\frac{1}{n}} \qquad (S3)$$

Its logarithmic form is：

$$\mathrm{In} q_e = 1/n \mathrm{In} C_e + \mathrm{In} K_F \quad (S4)$$

C_e—Equilibrium adsorption concentration, mmol L^{-1};

q_e—Equilibrium adsorption capacity，meq g^{-1};

K_F and 1/n —Adsorption constant，meq(1-1/n)/gL(1/n);

By plotting lnq_e against lnC_e, a straight line can be obtained. The adsorption constants KF and 1/n can be calculated from the slope and intercept of the line. The smaller the value of

1/n, the better the adsorption performance. Generally, n=1-10 is considered an easy adsorption process, while n<0.5 is a difficult adsorption process. By comparing these two constants, n and KF, the characteristics of different adsorbents can be assessed.

(3)Pseudo-second-order dynamics

The pseudo-second-order kinetic adsorption equation is as follows:

$$\frac{dq_t}{d_t} = k(q_e - q_t)^2 \qquad \text{(S5)}$$

q_e—Equilibrium adsorption capacity, mmol g^{-1};

q_t—Adsorption capacity at time t, mmol g^{-1};

k—Quasi-second-order rate constant, g $mmol^{-1}$ h^{-1};

The equation (5) is integrated, taking the initial conditions t=0, q_t=0 and t=t, q_t=q_t respectively; Formula (6) is obtained.

$$\ln(q_e - q_t) = \ln q_e - kt \qquad \text{(S6)}$$

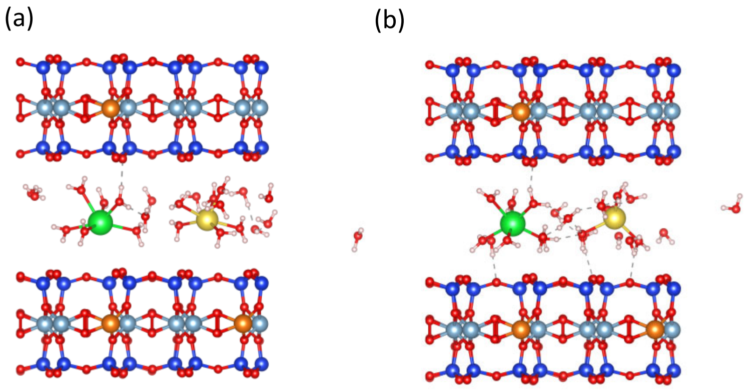

Figure S1. The formation of hydrogen bonds and water bridge between Sr^{2+} molecule and bentonite surface during an AIMD simulation (a and b).

Reference:

[1] Oleksiienko, O.; Wolkersdorfer, C.; Sillanpää, M. Titanosilicates in Cation Adsorption and Cation Exchange—A Review. Chem. Eng. J. 2017, 317, 570–585. [CrossRef]

[2] Cheng, Y.; Chuah, G.K. The synthesis and applications of-zirconium phosphate. Chin. Chem. Lett. 2020, 31, 307–310. [CrossRef]

[3] He, W.; Ai, K.; Ren, X.; Wang, S.; Lu, L. Inorganic layered ion-exchangers for decontamination of toxic metal ions in aquatic systems. J. Mater. Chem. A 2017, 5, 19593–19606. [CrossRef]

[4] Rogers, H.; Bowers, J.; Gates-Anderson, D. An isotope dilution–precipitation process for removing radioactive cesium from wastewater. J. Hazard. Mater. 2012, 243, 124–129. [CrossRef]

[5] Zakrzewska-Trznadel, G. Advances in membrane technologies for the treatment of liquid radioactive waste. Desalination 2013, 321, 119–130. [CrossRef]

[6] Ma, H.; Shen, M.; Tong, Y.; Wang, X. Radioactive wastewater treatment technologies: a review. Molecules 2023, 28(4), 1935. [CrossRef]

[7] Jia, F.; Li, J.; Wang, J.; Sun, Y. Removal of strontium ions from simulated radioactive wastewater by vacuum membrane distillation. Ann. Nucl. Energy 2017, 103, 363-368. [CrossRef]

[8] Shukla, A.; Parmar, P.; Saraf, M. Radiation, radionuclides and bacteria: An in-perspective review. J. Environ. Radioact. 2017, 180, 27–35. [CrossRef]

[9] Vanhoudt, N.; Vandenhove, H.; Leys, N.; Janssen, P. Potential of higher plants, algae, and cyanobacteria for remediation of radioactively contaminated waters. Chemosphere 2018, 207, 239–254. [CrossRef]

[10] Chen, K.; Chen, C.; Ren, X.; Alsaedi, A.; Hayat, T. Interaction mechanism between different facet TiO_2 and U (VI): Experimental and density-functional theory investigation. Chem. Eng. J. 2019, 359, 944–954. [CrossRef]

[11] J.A. Simon, S. Abrams, T. Bradburne, D. Lester, P. Storch, J. Remy, A. Dindal, PFAS Experts Symposium: Statements on regulatory policy, chemistry and analytics, toxicology, transport/fate, and remediation for per-and polyfluoroalkyl substances (PFAS) contamination issues, Remediation J. 2009, 29(4) 31–48. [CrossRef]

[12] Aytas, S.; Yurtlu, M.; Donat, R. Adsorption characteristic of U (VI) ion onto thermally activated bentonite. J. Hazard. Mater. 2009, 172(2-3), 667-674. [CrossRef]

[13] Hafizi, M.; Abolghasemi, H.; Moradi, M.; Milani, S. A. Strontium adsorption from sulfuric acid solution by Dowex 50W-X resins. Chin. J. Chem. Eng. 2011, 19(2), 267-272. [CrossRef]

[14] Abu-Nada, A.; Abdala, A.; McKay, G. Isotherm and Kinetic Modeling of Strontium Adsorption on Graphene Oxide. Nanomaterials *2021, 11, 2780*. [CrossRef]

[15] Naeimi, S.; Faghihian, H. Modification and magnetization of MOF (HKUST-1) for the removal of Sr^{2+} from aqueous solutions. Equilibrium, kinetic and thermodynamic modeling studies. Sep. Sci. Technol. 2017, 52(18), 2899-2908. [CrossRef]

[16] Lv, T. T.; Ma, W.; Zhang, D.; Zhang, T.; Tang, J. H.; Zeng, X.; Feng, M.L.; Huang, X. Y. Rapid and highly selective Sr^{2+} uptake by 3D microporous rare earth oxalates with the facile synthesis, high water stability and radiation resistance. Chem. Eng. J. 2022 435, 134906. [CrossRef]

[17] Zheng, B.; Yin, J.; Zhu, L.; Zhou, B.; Shen, H.; Harbottle, D.; Hunter, T.N.; Sheng, Y.; Zhu, Q.; Zhang, H. Thiol-rich and ion-imprinted alginate hydrogel as a highly adsorptive and recyclable filtration membrane for rapid and selective Sr (II) removal. Chem. Eng. J. 2023, 465, 142752. [CrossRef]

[18] Ali, M. M. S.; Abdel-Galil, E. A.; Hamed, M. M. Removal of strontium radionuclides from liquid scintillation waste and environmental water samples. Appl. Radiat. Isot. 2020, 166, 109357. [CrossRef]

[19] Liu, C.; Su, G.; Chen, T.; Liang, M.; Zhao, L.; Zhang, D. Preparation of sulfonylcalix [4] arene-loaded XAD-7 resin for strontium (II) adsorption. In IOP conference series: earth and environmental science. IOP Publishing 2019, 267(3), 032084. [CrossRef]

[20] Ogata, F.; Kobayashi, Y.; Uematsu, Y.; Nakamura, T.; Kawasaki, N. Zeolite produced from fly ash by thermal treatment in alkaline solution and its capability to adsorb Cs (I) and Sr (II) in aqueous solution. Yakugaku Zasshi-J. Pharm. Soc. Jpn. 2020 140(5), 729-737. [CrossRef]

[21] Marinović, S. S.; Ajduković, M. J.; Jović-Jovičić, N. J.; Mudrinić, T. M.; Nedić-Vasiljević, B. N.; Banković, P. T.; Milutinović-Nikolić, A. D. Adsorption of strontium on different sodium enriched bentonites. J. Serb. Chem. Soc. 2017, 82(4), 449-463. [CrossRef]

[22] Zhang, J.; Chen, L.; Dai, X.; Zhu, L.; Xiao, C.; Xu, L.; Zhang, Z.; Alekseev, E.V.; Wang, Y.; Zhang, C.; Zhang, H.; Wang, Y.; Diwu, j.; Chai, Z.; Wang, S. Distinctive two-step intercalation of Sr^{2+} into a coordination polymer with record high ^{90}Sr uptake capabilities. Chem 2019, 5(4), 977-994. [CrossRef]

[23] Zhang, L.; Wei, J.; Zhao, X.; Li, F.; Jiang, F. Adsorption characteristics of strontium on synthesized

antimony silicate. Chem. Eng. J. 2015, 277, 378-387. [CrossRef]
[24] Lee, C. P.; Tsai, S. C.; Wu, M. C.; Tsai, T. L. A study on removal of Cs and Sr from aqueous solution by bentonite–alginate microcapsules. J. Radioanal. Nucl. Chem. 2018, 318, 2381-2387. [CrossRef]
[25] Langmuir, I. The adsorption of gases on plane surfaces of glass, mica and platinum, J. Am. Chem. Soc. 1918, 40(9) 1361-1403. [CrossRef]
[26] Freundlich, H. Über die adsorption in lösungen. Z. Phys. Chem. 1907, 57(1), 385-470. [CrossRef]
